# Propranolol-loaded electrospun nanofibrous wound dressing: From fabrication and characterization to preliminary wound healing evaluation

**DOI:** 10.22038/ijbms.2021.57770.12857

**Published:** 2021-09

**Authors:** Sasan Zaeri, Fatemeh Karami, Majid Assadi

**Affiliations:** 1 Department of Pharmacology, School of Medicine, Bushehr University of Medical Sciences, Bushehr, Iran; 2 Department of Pharmacology, School of Pharmacy, Eastern Mediterranean University, Famagusta, North Cyprus; 3 Nuclear Medicine and Molecular Imaging Research Center, Bushehr University of Medical Sciences, Bushehr, Iran

**Keywords:** Electrospun nanofiber, Fibroblast, Mouse, Oxidative stress, Propranolol, Wound

## Abstract

**Objective(s):**

The wound healing potential of beta-blocker drugs such as propranolol (PNL) has recently attracted attention. To date, incorporation of PNL into electrospun nanofibrous wound dressing mats has not been tested as a novel topical drug delivery system. Presently, electrospun nanofibrous mats loaded with PNL were fabricated, and their physicochemical properties and wound healing activities were evaluated.

**Materials and Methods:**

Polyvinyl alcohol solutions containing 0, 2% or 4% (wt/vol) PNL were electrospun into mats, and the physicochemical properties and PNL release were evaluated. *In vitro* biocompatibility of selected PNL-loaded mats was tested in human foreskin fibroblasts and wound healing capability was evaluated in mouse skin wounds.

**Results:**

The 4% PNL mat had thin fibers (160 nm), convincing porosity (79.5%), and good hydrophilicity (swelling: 89.1%, water contact angle: 42.1°) with little degradability (14.2%). The release of PNL was not in bursts and was best explained by the Korsmeyer–Peppas equation (R2 = 0.96, n = 0.40), suggesting Fickian release. The viability of fibroblasts was 173% on day 5 of incubation with 4% PNL mats, indicating good mat biocompatibility. In vivo treatment for 14 days with 4% PNL mats resulted in wounds with a surface area of only 9% of the original wound area. These wounds had better histopathologic characteristics and were associated with less oxidative stress.

**Conclusion:**

The wound dressing fabricated with 4% PNL showed good potential for wound healing because of a favorable drug release profile from the nanofiber scaffold, and can be considered eligible for further clinical research.

## Introduction

Beta-adrenergic receptor blocking drugs, commonly known as beta-blockers, are well-established medicines in the treatment of cardiovascular disorders. They are generally classified as selective (beta-1) or nonselective (beta-1 and beta-2) beta-blockers depending on their affinity for beta-adrenoceptors (1). In addition to their well-known cardiovascular indications, evidence also exists to support their potential effectiveness in dermatology. Oral propranolol (PNL) is an FDA-approved beta-blocker used to treat infant hemangiomas, i.e., benign tumors of the skin vasculature (2). Moreover, PNL is reportedly effective in the treatment of ulcerated infantile hemangiomas by shortening the ulceration period (3). Beta-blockers have also shown promising effects on the wound healing process (4, 5). It is understood that stress induced by burns, traumatic injuries, or other sources plays a negative role in the wound healing process (6). In this connection, the presence of stress hormones such as epinephrine in the wound environment may hinder keratinocyte activation and wound re-epithelization (7). Of note, beta-2 receptors of the adrenergic system are well expressed on skin cells such as keratinocytes, and their inhibition by beta-blockers has been associated with greater keratinocyte migration and hence a better wound healing profile (8, 9). 

The normal wound healing process consists of four overlapping stages including hemostasis, inflammation, proliferation, and maturation or remodeling. Without the coordinated actions of all players in the wound healing process, good skin tissue regeneration cannot be achieved (10). Oxidative stress is an important parameter in wound healing, where it may play a negative role if not controlled. Although controlled levels of reactive oxygen species (ROS) are pivotal at the primary stage in the healing process – e.g., in the ability of phagocytic cells to destroy invading pathogens (11) – uncontrolled oxidative stress can have deleterious effects on the wound healing process (12, 13). It is well known that cellular oxidative stress may be produced, in part, by adrenergic receptor overactivation (14), a condition which is highly probable during burns and other skin injuries as a consequence of post-injury stimulation of the sympathetic system. Although several mechanisms of action have been proposed for the positive effects of beta-blockers in the wound healing process, there is as yet no consensus (5, 15). In light of the presence of beta-2 receptors in the skin tissue and the limiting role of oxidative stress in wound healing, it could be postulated that the potential effectiveness of beta-blockers in wound healing is mediated, in part, through a decrease in oxidative stress in the wound tissue. 

One major drawback of administering oral beta-blockers for dermatologic applications is their systemic adverse effects such as hypotension, changes in plasma glucose levels, and bronchoconstriction. Therefore, the use of topical formulations appears to be a more logical approach (16). However, conventional topical formulations such as gels, creams, and ointments might not fulfill all conditions for wound healing due to their intrinsic shortcomings, including the short duration of action and patient noncompliance (17). Instead, new alternative topical approaches such as nanofiber-based wound dressings have recently been investigated to document their particular characteristics in tissue engineering and wound healing (18, 19). Nanofibrous wound dressings have a number of unique physicochemical properties and highly resemble the extracellular matrix of the skin tissue (20). Among several techniques used to fabricate nanofibers, electrospinning of a polymer solution has been used most frequently because of the simplicity and high tunability of the process (21). 

**Figure 1:**
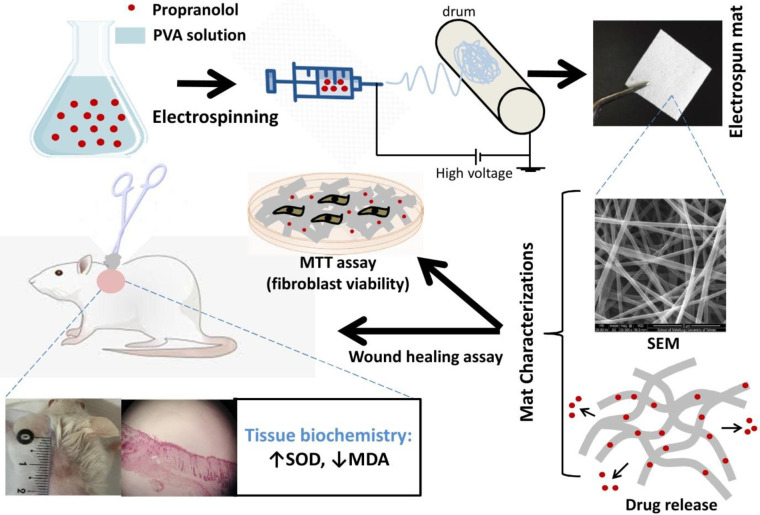
Graphical summary of the study illustrating fabrication, characterization, and examination of PNL-contained electrospun mats

The research reported here is based on the promising effects of beta-blockers in wound healing, which may in turn be associated with lower oxidative stress, and the documented benefits of nanofiber-based wound dressings. The objectives of the present study were (1) to fabricate nanofibrous mats loaded with PNL as a prototype beta-blocker, (2) to characterize the physicochemical properties of the mats, and (3) to assess their biological effects in wound healing. To this end, assays were done to evaluate *in vitro* drug release kinetics, biocompatibility with fibroblasts, the wound healing efficacy of the fabricated mat in mice, and the effects on oxidation-reduction (redox) status in wound tissues. A graphical summary of the study is shown in Figure 1.

## Materials and Methods

***Materials***

Propranolol hydrochloride and polyvinyl alcohol (PVA) (MW 72,000 Da) were supplied from Merck Co. (Germany). Glutaraldehyde (25% vol/vol) was from Fluka Co. (UK). Ketamine and xylazine injectable vials were from Alfasan Co. (the Netherlands). Double distilled water (dd H_2_O) was used throughout the study as the dissolving and diluting solvent.

***Electrospinning process***

The PVA solution was electrospun according to a previous method with some modifications (22). Briefly, 6% (wt/vol) PVA solution was made by dissolving PVA powder in dd H_2_O (80 °C) with gentle stirring for 3 hr. Mixtures of PNL at 0%, 2%, or 4% (wt/vol) concentration were prepared by adding the appropriate amounts of PNL to the PVA solution at 25 °C. Before electrospinning, the electrical conductivity of the above PVA solutions was determined using an electrical conductivity meter (WTW-Portable conductivity meter ProfiLine Cond 3310, Germany) at ambient temperature. Electrospinning was done with an electrospinner provided by Fanavaran Nano Meghyas Co. (Iran). A 5-ml syringe equipped with an 18-G needle was filled with each solution, and the solutions were injected (0.25 ml/hr) under 15–17 kV voltage with the needle tip 12 cm away from a rotating collector drum. The drum was covered with aluminum foil on which the electrospun fibrous mat was collected. The mat was subsequently peeled off and dried under a vacuum for 6 hr. The fabricated mats prepared from PVA solutions containing 0%, 2%, or 4% (wt/vol) PNL will be referred to hereafter as neat PVA, PVA/PNL 2%, or PVA/PNL 4% mats, respectively. The fabricated mats were treated with diluted glutaraldehyde (10% vol/vol) vapor at 25 °C for 12 hr so that fibers became cross-linked and resistant against sudden dissolution and/or disintegration. Unreacted glutaraldehyde was removed by briefly rinsing the mats in dd H_2_O, and the mats were subsequently dried under a vacuum. 

***Fiber size and morphology***

Scanning electron microscopy (SEM) (CamScan-MV2300, USA) was used to determine the size and morphology of the fibers. Briefly, gold-coated thin mats (1 × 1 cm) were subjected to a voltage of 20 kV, and the SEM images were imported into ImageJ Software v. 1.47 (National Institutes of Health, USA) to determine fiber size. The average fiber size of each mat was calculated by measuring the thickness of 50 to 100 randomly chosen fibers in the SEM image.

***Swelling, water contact angle, and weight loss tests***

Swelling was tested by immersing mats in phosphate-buffered saline (PBS) (pH 7.4, 25 °C) for one hour. The next simple equation was used to calculate percent swelling in each mat:

$\mathrm{Swelling}\left(\%\right)=\frac{M-M_{i}}{M_{i}}\times 100$

Equation 1

where *M* is the mat weight after immersion in the PBS solution, and *M*_i_ is the initial weight of the mat in the dry state before immersion.

The water contact angle test was used to estimate the wettability of the mats. For this simple test, a contact angle goniometer (OCA 20, Germany) was connected to a charged-coupled device (CCD) camera. Briefly, 10 µl deionized water was put on the mat surface and photographed to determine the contact angle with the surface.

To document mat resistance against rapid disintegration, a gravimetric assay called the weight loss test was used (23, 24). A mat sample of approximately 50 mg was weighed initially and immersed in PBS buffer (20 ml, pH 7.4, 37 °C) for three days. Then the mat was taken out, washed gently in doubly distilled H_2_O, and vacuum-dried. Weight loss (%) of the mat after 72 hr was calculated with the following formula: 

$\mathrm{Weight loss}\left(\%\right)=\frac{M_{72}-M_{i}}{M_{i}}\times 100$

Equation 2

where *M*_i_ is the initial weight of the mat (dry weight before immersion in PBS) and *M*_72_ is its weight after 72 hr (dry weight after immersion in PBS).

***Porosity test***

The following theoretical method was used to estimate the porosity of the fabricated mats (23, 25): 

$\mathrm{Porsity}\left(\%\right)=(1-\frac{\rho_{m}}{\rho_{0}}\times 100$

Equation 3

In this formula, *ρ*_m_ is the apparent calculated density and *ρ*_0_ is the density of the PVA powder in bulk form [*ρ*_0__ (PVA)_ = 1.3 g/cm^3^]. To find *ρ*_m_, mats were put on each other layer by layer (400 µm thick) and weighed precisely (ρ_m_ = mass/volume of stacked mats).

***Fourier transform infrared spectroscopy (FTIR)***

FTIR (Perkin Elmer, Frontier, USA) was utilized to verify the chemical composition of PVA and PNL in the electrospun fibers and to check for any probable molecular interactions between the ingredients. Briefly, pure PVA, pure PNL, or the powdered PNL-loaded mat was mixed separately with potassium bromide at 1:100 (wt/wt), and the FTIR spectra were obtained through 400 to 4000 cm^-1^.

***Study of PNL release from mats***

PNL-contained mats in PBS (15 ml, pH = 7.4, 37 °C) were gently stirred for 1, 2, 3, 6, 12, 24, 72, 96, and 120 hr. At each time point, an aliquot of 2 ml of the solution was removed to estimate PNL concentration at 291 nm (Shimadzu UV-1100) based on a previous standard curve (PNL 0-100 µg/ml). After each sample was withdrawn, the same amount of PBS (2 ml) was replaced to lost volume. 

The release mechanism was also explored using a number of release kinetics models including zero order, first order, Higuchi, and Korsmeyer–Peppas (Table 3). Of note, the last model is well known in studies designed to characterize release mechanisms from polymeric matrices (26).

***In vitro fibroblast viability assay***

The MTT (3-(4, 5-dimethyl-2-thiazolyl)-2, 5-diphenyl-2H-tetrazolium bromide) assay was used to investigate how compatible the fabricated mats were with human fibroblasts (HNFF-P18 cell line, Pasteur Institute Cell Bank, Iran). In brief, HNFF-P18 cells were cultured in DMEM which contained 10% (vol/vol) fetal bovine serum (FBS), penicillin (100 U/ml), and streptomycin (100 μg/ml), and were then incubated in a 5% CO_2_ incubator at 37 °C. After the mats were wetted with 250 μl DMEM in a 24-well multiplate, cells from passage four were seeded on them at a density of 2.5 × 10^4^ cells per well and incubated for 1, 3, and 5 days. Then, the cells-mats were incubated with MTT solution (5 mg/ml) for extra 4 hr. Absorbance in each well was read at 570 nm with a microplate reader (Power waveX, Bio-Tek Instruments, USA). Equation 4 below was used to calculate percent fibroblast viability, with *A*_s_, *A*_c_, and *A*_b_ representing sample (mat + cell), control (cell only), and blank absorbance, respectively. 

$\mathrm{Fibroblast visibility}\left(\%\right)=\frac{A_{s}-A_{b}}{A_{c}-A_{b}}\times 100$

Equation 4

***Wound healing study in mice***

*Wound model*

Mice weighing 25–30 g were provided by the animal facility of Bushehr University of Medical Sciences (BPUMS), Iran. One week before the wound experiments started, the mice were moved to their new housing for familiarization. All animals were housed in standard cages with 2 or 3 animals per cage under light-dark cycles (12 hr) at 25 °C and had free access to food pellets and water. Permission for the animal study was obtained from the Ethics Committee of BPUMS, and the animals were handled in accordance with globally accepted guidelines for working with experimental animals. 

After mice were anesthetized with intraperitoneal ketamine (60 mg/kg) and xylazine (10 mg/kg), the interscapular area was shaved and disinfected with alcohol, and then a circular wound with a diameter of one centimeter was made using a biopsy punch. All wounds were full-thickness, but the muscles beneath the skin were left intact.

*Mouse model of in vivo wound healing*

Mice (total number: 40) were randomly allocated to four groups that received one of the topical treatments: group 1, saline; group 2, 4% PNL gel; group 3, neat PVA mat; group 4, PVA/PNL 4% mat. We included the 4% PNL gel group to compare the effects of PNL on wound healing in two different pharmaceutical vehicles: nanofibers versus gel. The 4% PNL gel consisted of a gel-like PVA solution containing 4 g PNL per 100 ml of solution. All topical preparations were disinfected with ultraviolet-C light for 30 min before use. Treatments were applied on the wounds once daily, and the wounds were bandaged with simple sterile gauze. Five animals in each group received treatment for 7 days and the other 5 animals continued to receive treatment until day 14. 

For macroscopic studies, the wounds were photographed with a digital camera immediately after they were made (day 0) and subsequently on days 3, 7, and 14 post-surgery. For scale, a ruler was placed adjacent to each wound when it was photographed. The animals were killed either 7 or 14 days after surgery by high-dose ketamine/xylazine injection in the heart, and wounds with the surrounding normal skin were excised cut vertically into two pieces: one to be stained for histopathologic analysis and the other to be stored at −80 °C for biochemical analysis. 

*Macroscopic evaluation of wounds*

Wound areas were processed with ImageJ Software v. 1.47. To compensate for variations in the initial size of the wounds, the calculated wound size on days 3, 7, or 14 was normalized to the size of the same wound on day 0 (surgical day) and reported as wound area (%). The following equation was used to calculate the wound area: 

$\mathrm{Wound area}\left(\%\right)=\frac{A_{d}}{A_{0}}\times 100$

Equation 5

where* A*_0_ and *A*_d_ are wound areas on day 0 and the selected day, respectively. 

*Microscopic histopathologic measurements*

The effects of treatments on histopathologic parameters of wound healing were investigated with hematoxylin-eosin (H&E) and Masson’s trichrome stainings. Briefly, the explanted wound tissues were sectioned vertically, fixed in 3.7% formalin, and embedded in paraffin. Then 5-µm sections of tissue samples were cut and stained according to routine protocols for H&E and Masson’s trichrome staining. The microscopic parameters recorded were level of re-epithelization, numbers of polymorphonuclear (PMN) leukocytes, numbers of fibroblasts, and collagen density. These parameters were scored by an expert pathologist based on a scaling system reported previously (27) (Table 1).

*Biochemical assay to estimate oxidative stress in wound tissues*

The skin wound tissue (50 mg) was homogenized in PBS buffer (pH = 7.4, 4 °C) for 60 sec with a mechanical homogenizer (Eberbach Corporation, Ann Arbor, MI, USA) and centrifuged at 4000 rpm for 20 min to obtain supernatants for the assay. The Bradford method was used to measure total protein content in the samples, with bovine serum albumin as the standard (28). Two major indices of oxidative stress in tissues were determined: lipid peroxidation and superoxide dismutase (SOD) activity. In the lipid peroxidation assay, which was based on the thiobarbituric acid reactive substances (TBARS) method, the level of malondialdehyde (MDA) as the main by-product of lipid peroxidation was determined spectrophotometrically at 532 nm and reported in nmol/mg protein. The SOD enzyme is mainly responsible for the conversion of superoxide anions to less reactive hydrogen peroxide. In the SOD activity assay, the remaining unreduced superoxide anions react with a dye. The absorbance of the product is measured at 440 nm. This indirectly indicated the level of SOD activity in tissues (unit/mg protein). The two assays were done with commercially available MDA and SOD assay kits (Abnova, Taiwan). 

***Statistical analysis***

The data are reported as the mean ± standard deviation from at least 3 assays. Wound area, MDA, and SOD measurements were analyzed by one-way ANOVA with the Tukey *post hoc* test. For microscopic measurements which produced scored data, the nonparametric Kruskal–Wallis test with subsequent pairwise comparisons was used. *In vitro* viability data were analyzed with the independent samples *t*-test. SPSS^®^ Software (v.21) was used for data analysis (significance level: *P*<0.05).

## Results

***Fiber size distribution and morphology***

**Figure 2:**
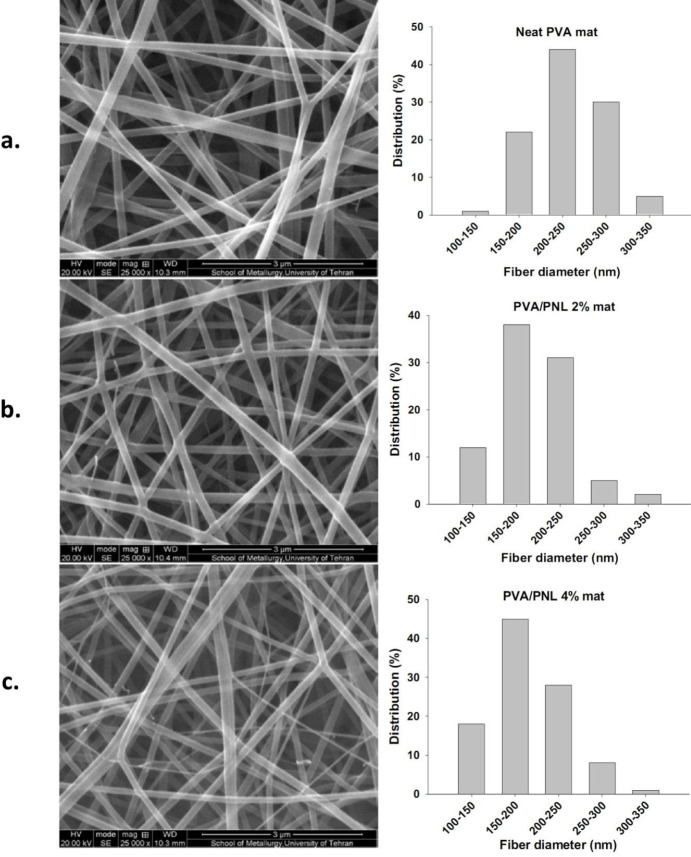
Representative SEM photos (left) and the corresponding size distribution graphs (right) of fibers with different compositions. Scale bars in all photos: 3000 nm

Representative SEM images of the electrospun fibers and their corresponding size distributions are shown in Figure 2. Fibers were thin and uniform throughout the range of PNL concentrations from 0% to 4%. The average thickness of fibers in the neat PVA mat was 193 ± 47 nm, whereas the PVA/PNL 2% and 4% mats had lower mean fiber sizes of 175 ± 38 nm and 160 ± 50 nm, respectively. This showed that fiber diameter became smaller when higher amounts of PNL were used in the solution.

***Hydrophilicity, degradability, and porosity of mats***

The results for hydrophilicity, degradability (weight loss), and porosity of mats are summarized in Table 2. In the swelling assay, the neat PVA mat showed up to 87.3% swelling, and the PNL-containing mats showed very similar values. These results indicated that the extent of swelling was not affected by the presence of PNL. Similarly, water contact angle measurements disclosed no substantial differences between the neat and PNL-loaded mats. Taken together, the findings from swelling and contact angle assays indicated that the addition of PNL to nanofibers did not lead to noteworthy changes in hydrophilicity, although the mats can generally be considered hydrophilic. Findings from weight loss tests showed that weight loss values of PVA/PNL 4% mat, PVA/PNL 2% mat, and neat PVA mat were 14.2%, 14.8%, and 15.1%, respectively in the aqueous medium after 72 hr. All electrospun mats were found to be porous, a good feature for a wound dressing. However, porosity in the PVA/PNL 4% mat was 4.1% higher than the PVA/PNL 2% mat and 10.7% higher than the neat PVA mat. These results showed that the higher concentration of PNL increased the porosity of the mats, which may be associated with the thinner fiber diameter in the PVA/PNL 4% mat.

***FTIR***

**Figure 3:**
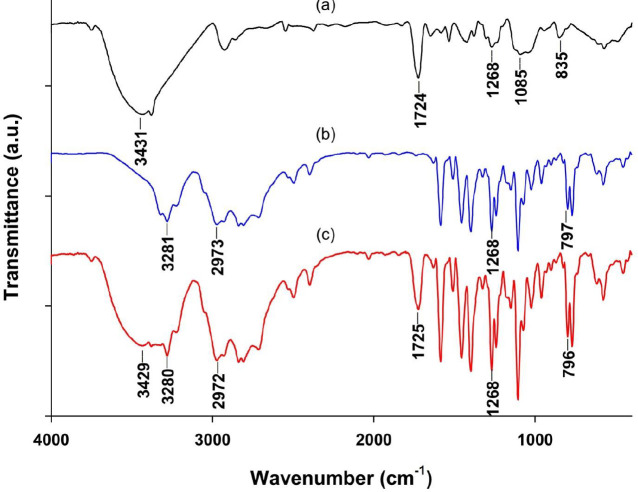
FTIR spectra of (a) PVA powder, (b) pure PNL powder, and (c) PVA/PNL mat. The main peaks for each material are indicated

PVA: polyvinyl alcohol; PNL: propranolol

Figure 3 illustrates spectra of PVA powder, PNL powder, and PVA/PNL mat. The peaks at 835, 1085, and 1268 cm^-1^ in the PVA spectrum (Figure 3a) corresponded to C-C stretching, C-O stretching, and C-H bending vibrations, respectively (29). The peak at 1724 cm^-1^ was the result of stretching vibration in the terminal acetyl group in PVA. Stretching of the alcohol group (-OH) in PVA produced a characteristic wide peak centered around 3431 cm^-1^. The main characteristic peaks for pure PNL (Figure 3b) were seen at 2973 cm^-1^_, _corresponding to a secondary amine group, at 3281 cm^-1^ due to stretching of secondary -OH, at 1268 cm^-1^ due to stretching of an aryl alkyl ether group, and at 797 cm^-1^, which was attributed to alpha-substituted naphthalene in the PNL molecule (30). The spectrum for the electrospun PVA/PNL mat (Figure 3c) displayed the same peaks as the pure materials, indicating that no major interactions took place between PVA and PNL molecules in the electrospun fibers.

***Release profile of PNL***

**Figure 4:**
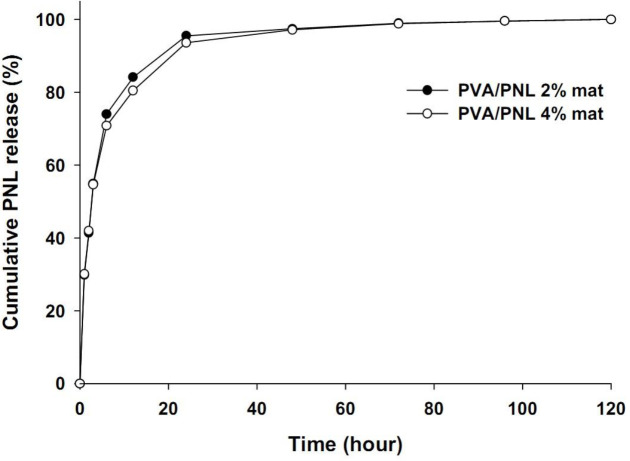
PNL release (cumulative %) from the PVA/PNL 2 and 4% mats in the PBS buffer solution (pH = 7.4, 37 °C) within a time span of 120 hr

PVA: polyvinyl alcohol; PNL: propranolol

The cumulative PNL release dynamics from PVA/PNL 2% and 4% mats in BPS are shown in Figure 4. The shape of the two release curves was generally similar for both formulations, and nearly overlapping with each other indicating that the amount of PNL in the mats did not influence release rates. Compared with total release after 120 hr, about 95% of drug release from the PVA/PNL 2% mat and 93% of drug release from the PVA/PNL 4% mat occurred within the first 24 hr, and release thereafter was very slow, as shown by the plateau in the release curves. Maximum concentrations of PNL in the plateau stage were 25.8 µg/ml in PVA/PNL 2% and 53.6 µg/ml in PVA/PNL 4% mats.

As shown in Table 3, different release kinetics models were used to determine the mechanism of PNL release from the fabricated mats. The data of PVA/PNL 2 and 4% mats showed the highest fit in the Korsmeyer–Peppas model (R^2^ = 0.96), although fit with the Higuchi model was also very good (R^2^ = 0.94 and 0.95). The diffusion exponent used to predict the release mechanism in Korsmeyer–Peppas equation, was below 0.5 for mats with both concentrations of PNL, suggesting a Fickian diffusion mechanism for PNL release.

***In vitro*** **biocompatibility of mats with human fibroblasts**

**Figure 5:**
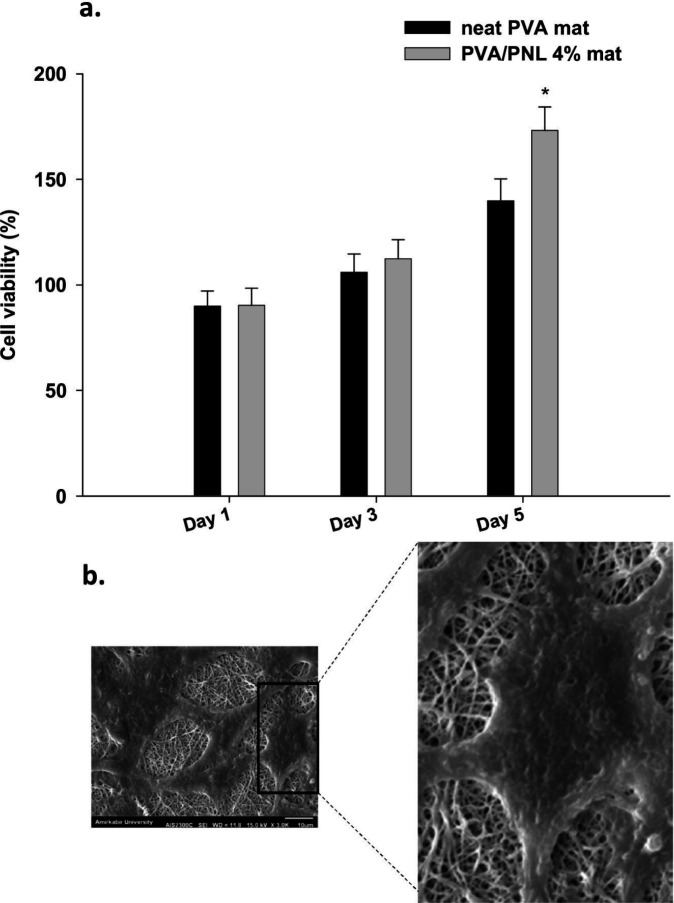
(a) Viabilities of fibroblasts after incubation with neat or PVA/PNL 4% mat for different periods of time. Bars indicate mean ± standard deviation (n=5). (b) A representative SEM photo of fibroblast proliferation on a PVA/PNL 4% mat on day 5 of incubation. The cells attached to the scaffold appeared to be fully elongated. The scale bar in the original SEM image represents 10 µm. Asterisk (*) mark: significant difference in viability (%) in comparison with neat PVA mat (*P*<0.05)

PVA: polyvinyl alcohol; PNL: propranolol

The effects of neat PVA and PVA/PNL 4% mats on fibroblasts viability in the MTT assay are shown in Figure 5. Percent viabilities of cells exposed to the neat PVA and PVA/PNL 4% mats did not differ significantly on the first (*P*=0.93) or third (*P*=0.28) day of incubation. However, cell viability was significantly greater with the PVA/PNL 4% mat on day 5 compared with the neat PVA mat (*P*=0.001) (Figure 5a). These results show that the fabricated mats were not only safe to the human fibroblasts but also promoted cell survival. Figure 5b is an SEM microphotograph of fibroblasts on the surface of PVA/PNL 4% mat on day 5. The cells attached to the nanoscaffold and adopted an elongated shape. This morphological change suggests effective interaction between cells and the nanofibrous scaffold, and may also favor fibroblast migration. The observed cellular morphology was also in line with the findings of the MTT assay, in which cell viability was greatest on the fifth day.

***Macroscopic (wound area) findings ***

**Figure 6:**
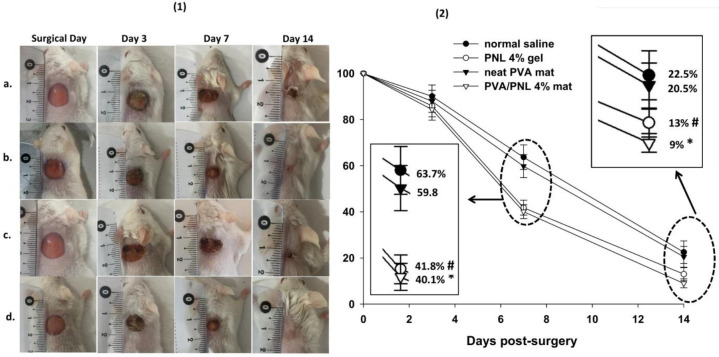
(1) Representative photographs of skin wounds after different periods of treatment with (a) saline, (b) PNL 4% gel, (c) neat PVA, or (d) PVA/PNL 4% mat. (2) The corresponding graph shows trends with time in wound areas. Statistically significant differences (P<0.05) between the PVA/PNL 4% mat and normal saline are shown by asterisk (*) mark. In addition, statistically significant differences between the PNL 4% gel and normal saline are shown by (#) mark

PVA: polyvinyl alcohol; PNL: propranolol

Figure 6 shows photographs of wounds treated in different groups on different post-surgical days. To illustrate changes in wound areas with time, the corresponding mean values for wound area are also graphed next to the wound photos. In all experimental groups, wound size tended to decrease from day 0 to day 14 although the slope of this trend differed among groups. The smallest wound area was observed in the PVA/PNL 4% mat group (9.0 ± 1.9%) on day 14 post-surgery, and the largest area was seen in the normal saline group (90.1±4.9%) on day 3 post-surgery. Between-group analysis of wound areas with one-way ANOVA revealed no significant differences on day 3 (F = 1.96, df = 3, *P*=0.14). However, the differences became statistically significant on day 7 (F = 51.96, df = 3, *P*<0.001) and remained significant on day 14 (F = 20.02, df = 3, *P*<0.001) post-surgery. Analysis of data showed that the PVA/PNL 4% mat and the PNL 4% gel resulted in smaller wound areas by 37% (*P*<0.001) and 34% (*P*<0.001), respectively, comparing with control saline on day 7. The PVA/PNL 4% mat and the PNL 4% gel were again associated with significantly smaller wounds by 60% (*P*<0.001) and 42% (*P*<0.001), respectively, on day 14. More changes in wound area during treatment with the PVA/PNL 4% mat or the PNL 4% gel occurred on day 14 compared with day 7.

***Microscopic (histopathologic) findings in wound healing***

**Figure 7:**
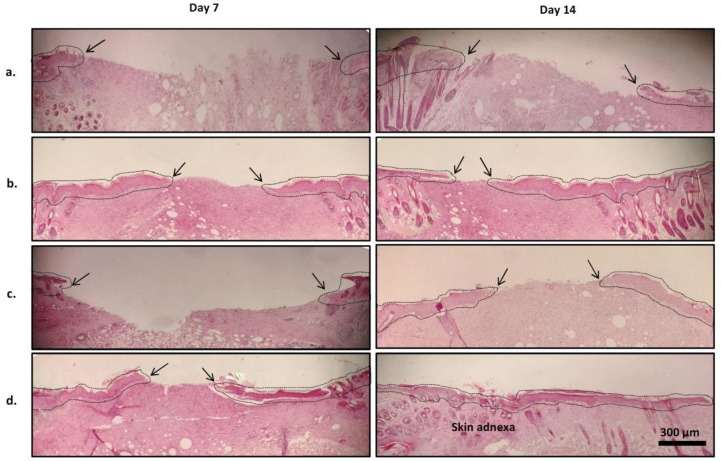
Representative H&E images of wounds after daily topical treatment with (a) saline, (b) PNL 4% gel, (c) neat PVA, or (d) PVA/PNL 4% wound dressing. Dashed lines encompass re-epithelization areas. Arrows (→) show wound re-epithelialization edges

PVA: polyvinyl alcohol; PNL: propranolol

**Figure 8:**
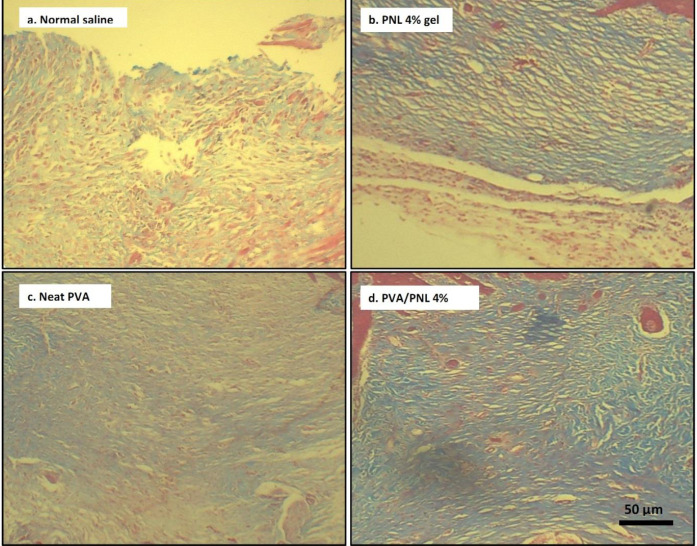
Representative Masson-trichrome images of wounds on day 14 post-surgery. The blue color represents stained collagen fibers. Collagen fibers are scarce in saline-treated wounds, moderate in the neat PVA group but more abundant in PNL-bearing treatments including PNL 4% gel and especially the PVA/PNL 4% mat

PVA: polyvinyl alcohol; PNL: propranolol

Micrographs of representative H&E-stained wound tissues were displayed in Figure 7. Figure 8 shows Masson’s trichrome-stained tissues after 14 days of treatment. In the normal saline group, wound re-epithelization was scarce on day 7 but increased somewhat on day 14 post-surgery (Figure 7a). The wound stroma was inflammatory on day 7. On day 14, fibroblasts and collagen fibers were scarce, presented by weak blue in Masson’s trichrome image (Figure 8a). The histological appearance of wounds in the PVA group was identical to saline-treated wounds 7 days after surgery, but on day 14 post-surgery the healing profile was slightly better with regard to re-epithelization and collagen synthesis (Figures 7c and 8c). Wounds treated with PNL 4% gel exhibited more re-epithelization compared with the previous groups (Figure 7b). Also, the more intense blue color in Masson’s trichrome image in this experimental group indicated high fibroblast content and hence the presence of high collagen fibers (Figure 8b). The histopathologic findings in the last group of wounds, i.e., those receiving PVA/PNL 4% mat were better than in the PNL 4% gel group. More complete re-epithelization and a denser fibrotic background were seen after 14 days of treatment in the PVA/PNL 4% mat-treated wounds, which were indicative of greater collagen accumulation (Figures 7d and 8d). Unlike the wounds in other experimental groups, PVA/PNL 4% mat-treated wounds showed evidence of the development of new skin adnexa on day 14 post-surgery. Although the recovery of skin adnexa was not complete, this finding was further evidence of the superiority of the PVA/PNL 4% mat in promoting wound healing compared with other treatments.

Statistical analysis gave a quantitative measure on the aforementioned histopathologic results (Table 4). Between-group differences were significant for the re-epithelization parameter (χ^2^ = 11.67, df = 3, *P*=0.009) on the seventh day. Wounds treated with PNL-containing formulations, i.e., the PVA/PNL 4% mat (*P*=0.023) and the PNL 4% gel (*P*=0.039), scored significantly higher than wounds treated with normal saline. Although the mean scores for PMNs were lower and the mean scores for fibroblasts were higher in the PVA/PNL 4% mat and PNL 4% gel groups than in the normal saline group, these differences were not statistically significant on day 7 post-surgery. Analysis of the data for day 14 post-surgery, however, showed significant between-group differences in mean scores for all parameters including re-epithelization (χ^2^ = 12.40, df = 3, *P*=0.006), PMNs (χ^2^ = 11.42, df = 3, *P*=0.010), fibroblasts (χ^2^ = 10.64, df = 3, *P*=0.014), and collagen (χ^2^ = 12.93, df = 3, *P*=0.005). Pairwise comparisons showed that on day 14, wounds in PVA/PNL 4% group scored better in terms of wound healing than wounds treated with normal saline on all histopathological parameters. Overall, mean scores for re-epithelization, fibroblasts, and collagen were highest, and mean scores for PMNs were lowest, in the PVA/PNL 4% mat group compared with all other groups both on day 7 and day 14 post-surgery. These histopathologic findings indicated a better wound healing process during treatment with the PVA/PNL 4% mat.

***Oxidative stress in wound tissues***

**Figure 9:**
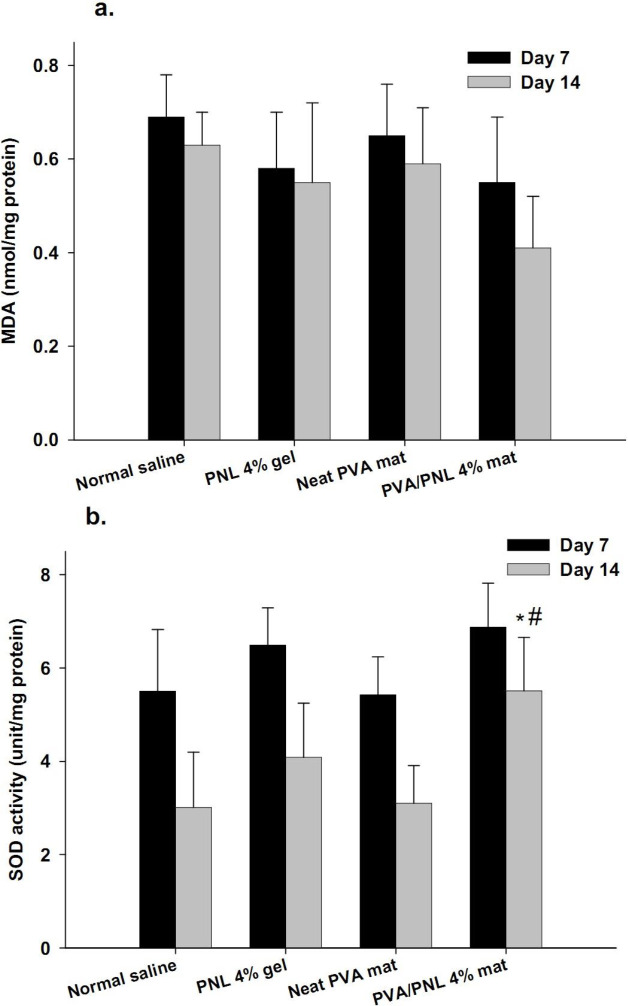
Means ± standard deviations of (a) MDA and (b) SOD levels in wounds either on day 7 or 14 post-surgery. (n = 4–5/group)

MDA: malondialdehyde; SOD: superoxide dismutase

Figure 9 summarizes the oxidative stress findings in wounds in terms of MDA and SOD activity levels on days 7 and 14 post-surgery. The MDA levels did not differ significantly among experimental groups on day 7 (F = 1.37, df = 3, *P*=0.28), although the levels in PVA/PNL 4% mat-treated wounds were 20.2% lower than normal saline group. After 14 days, MDA levels were generally lower than on day 7 post-surgery in all experimental groups, but again no significant between-group differences were seen (F = 2.97, df = 3, *P*=0.065). The greatest reduction in MDA levels on day 14 post-surgery was observed in the PVA/PNL 4% mat, where this activity was 34.9% smaller than saline (*P*=0.057). Although the *P*-value of 0.057 was not statistically significant, it approached significance. The SOD activity levels on day 7 post-surgery were not significantly affected by the treatments compared with normal saline (F = 2.61, df = 3, *P*=0.089), although the levels in wounds treated PNL-bearing topicals were higher. Analysis of day-14 data, however, showed that SOD levels were significantly different among groups (F = 5.28, df = 3, *P*=0.011). *Post hoc* analysis disclosed that these larger differences arose from the high SOD levels in PVA/PNL 4% group: 83.0% (*P*=0.014) and 77.7% (*P*=0.025) higher than saline and neat PVA groups, respectively.

PNL: propranolol

**Table 1:** Description of the scale used to score wounds in high-power microscopic fields

**Scale**	**Re-epithelization**	**PMNs (No.)**	**Fibroblasts (No.)**	**Collagen** ^*^
0	Absent	Absent	Absent	Absent
1	Covering <50% of the wound	<5	1-10	Scarce
2	Covering >50% of the wound	5-15	10-20	Moderate
3	Covering 100% of the wound with keratinization	>15	>20	Abundant

* Based on the intensity of blue staining in tissues prepared with Masson’s trichrome staining

**Table 2:** Estimation of hydrophilicity, degradability, and porosity of the fabricated nanofibrous mats

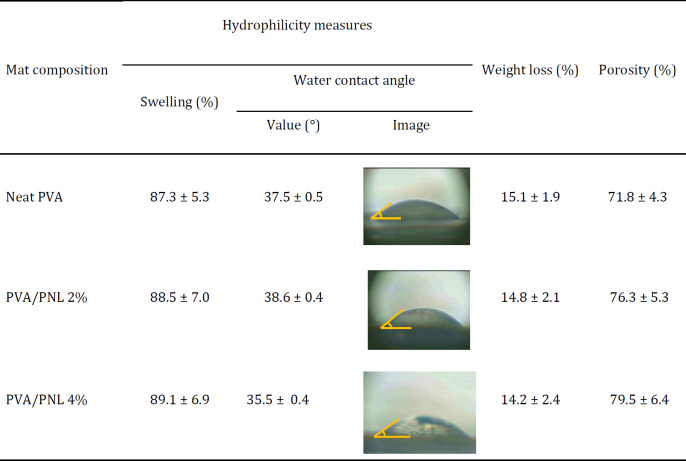

PVA: polyvinyl alcohol; PNL: propranolol

**Table 3:** Different mathematical kinetic models used to fit the drug release data for PVA/PNL 2% and 4% mats

Model	General equation	Resolved equation	
		PVA/PNL 2% mat	PVA/PNL 4% mat
Zero order	$C_{t}/C_{\infty }=k_{0}t$	$C_{t}/C_{\infty }=0.55t$ R^2^ = 0.48	$C_{t}/C_{\infty }=0.52t$ R^2^ = 0.47
First order	$1-(C_{t}/C_{\infty })=e^{-k_{1}t}$	$1-(C_{t}/C_{\infty })=e^{-0.026t}$ R^2^ = 0.89	$1-(C_{t}/C_{\infty })=e^{-0.01t}$ R^2^ = 0.87
Higuchi	$C_{t}/C_{\infty }={k_{H}t}^{0.5}$	$C_{t}/C_{\infty }={25.18t}^{0.5}$ R^2^ = 0.94	$C_{t}/C_{\infty }={23.86t}^{0.5}$ R^2^ = 0.95
Korsmeyer-Peppas	$C_{t}/C_{\infty }={k_{P}t}^{n}$	$C_{t}/C_{\infty }={1.49t}^{0.43*}$ R^2^ = 0.96	$C_{t}/C_{\infty }={1.49t}^{0.40*}$ R^2^ = 0.96

C_t_ and C_∞_: drug concentration at time t and plateau level (maximum concentration), respectively

k_0_, k_1_, k_H_, and k_P_: rate constants

n: diffusion exponent

PVA: polyvinyl alcohol; PNL: propranolol

**Table 4:** Average scores of pathologic findings in wounds on day 7 and day 14 post-surgery

Treatment	Day-7 mean rank^#^					Day-14 mean rank^#^			
	Re-epithel.	PMN	Fibroblast			Re-epithel.	PMN	Fibroblast	Collagen
Normal saline	8.4	20.0	10.6		4.2		16.8	5.1	5.1
PNL 4% gel	18.8^*^ (*P*=0.039)	11.9	17.0		11.25		7.5^*^ (*P*=0.048)	10.8	14.2
Neat PVA mat	12.3	14.8	15.2		8.9		11.7	8.4	7.7
PVA/PNL 4% mat	20.0^*^ (*P*=0.023)	9.7	15.8		15.9^*^ (*P*=0.003)		6.0^*^ (*P*=0.013)	15.8^*^ (*P*=0.009)	16.2^*^ (*P*=0.011)

# Mean scores generated with the Kruskal–Wallis test for pathologic features (n = 4–7/group)

* Statistically different versus saline group (*P*<0.05)

PVA: polyvinyl alcohol; PNL: propranolol

**Figure 10:**
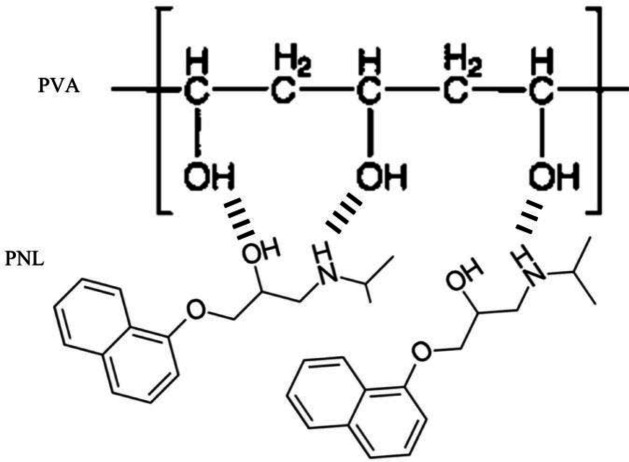
Schematic illustration of possible hydrogen bonding sites between PVA and PNL molecules. PVA: polyvinyl alcohol, PNL: propranolol

**Figure 11:**
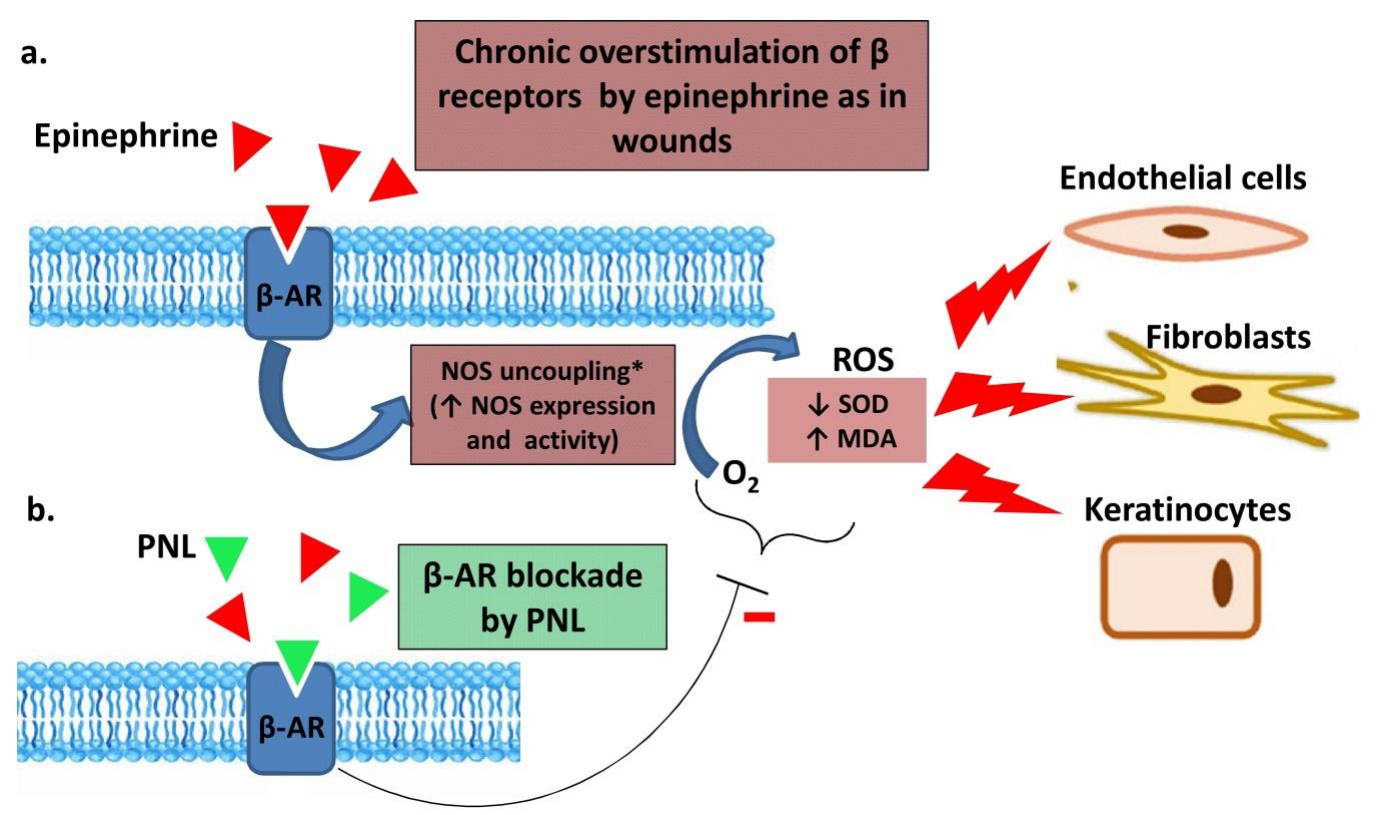
Schematic presentation of the proposed propranolol mechanism in wound healing in terms of lowering oxidative stress. (a) Endothelial cells, fibroblasts, and keratinocytes express beta-adrenergic receptors (β-AR). Chronic overstimulation of β-ARs by epinephrine –as in chronic stressful wounds- produces ROS. This might be mediated through uncoupling of nitric oxide synthase (NOS). ROS potentially hinders wound healing by negative effects on fibroblast and keratinocyte migration, collagen production, re-epithelization, and angiogenesis. (b) β-AR blockade by PNL may reverse the above negative effects of ROS. * NOS uncoupling was adopted from a previous work by Toblli *et al*. (41)

ROS: reactive oxygen species; PNL: propranolol

## Discussion

In this study, a PNL-loaded nanofibrous wound dressing was electrospun and tested for a number of physicochemical characteristics and biological efficacy in wound healing, both *in vivo* and *in vitro*. Electrospinning of PNL-containing PVA solutions yielded thin fibers with decreasing thickness from 193 nm to 160 nm as the PNL concentration increased from 0% to 4%. This finding may reflect changes in solution polarity upon addition of PNL. Two polar chemical groups in this drug, i.e., hydroxyl and amine, may render the solution more conductive in the strong electrical field used for electrospinning, and hence result in lower fiber thickness (31). In an effort to test this hypothesis, the electrical conductivity of the neat PVA, PVA/PNL 2%, and PVA/PNL 4% solutions were measured before electrospinning. The electrical conductance values of the above solutions were 717 ± 0.034, 6144 ± 0.301, and 7948 ± 0.128 µS/cm, respectively. Hence, as PNL concentration increased, the electrical conductance of the solution increased too. This confirmed the above notion about the relationship between solution polarity and fiber diameter. 

The shapes of the cumulative release curves for both PVA/PNL 2% and 4% mats were almost identical, indicating that the amount of PNL did not influence the pattern of drug release from the mats. However, the PVA/PNL 4% mat produced higher absolute concentrations of PNL in the release medium than the 2% formulation at equivalent time points. This may be just related to the higher PNL content in PVA/PNL 4% mat. Accordingly, electrospun polycaprolactone mats loaded with tetracycline at a higher concentration (5%) were found to release more tetracycline than mats with lower tetracycline content (2%) (32). The initial rapid release of PNL from both mats during the first few hours was probably associated with PNL dissolution from the surface and near-surface parts of nanofibers (33). Release of the inner PNL probably required the penetration of water into deeper parts of nanofibrous mats, resulting in a gradual release of PNL as shown by the subsequently slower rate of release (Figure 4). About 95% of PNL release from the PVA/PNL 2% mat and 93% of PNL release from the PVA/PNL 4% mat occurred within the first 24 hr. This means that most of the PNL were released to the wound tissue within 24 hr. 

Fitting our drug release data to different kinetics models revealed that the Korsmeyer–Peppas model explained PNL release most appropriately. Diffusion exponents for the PVA/PNL 2% mat (n = 0.43) and the PVA/PNL 4% mat (n = 0.40), both of which were less than 0.5, a Fickian release mechanism can be posited. In this case, transport of the drug into the solvent medium is dependent mainly on the concentration gradient of the drug and on polymer swelling due to solvent penetration in the matrix (34). Aside from superficial PNL, which is readily diffusible into the solution, entry of the solvent into the polymer fibers is also critical to dissolve and extract the drug in the Fickian diffusion mechanism. However, given that the PVA matrix naturally tends to swell with time upon exposure to an aqueous solvent, PNL release may be hindered later because the swollen polymer itself may act as a barrier to diffusion, ending up with more delayed-release after the initial fast release (35). The hydrogen bonding between the PVA backbone and PNL as proposed in Figure 10 may be another important parameter that might favor hindering the PNL release from the polymeric matrix.

The fibroblasts viability for PVA/PNL 4% mat increased with time. This indicated cell biocompatibility with PVA/PNL 4% mat. In addition, the PVA/PNL 4% mat yielded significantly higher viability on day 5 of incubation, suggesting that the presence of PNL was responsible for the observed difference. Marques *et al*. also showed that exposure of human dermal fibroblasts to a nanoemulsion containing PNL was associated with good cell viability through a relatively wide range of PNL concentrations (36). Further evidence of biocompatibility comes from an earlier study in which antagonism of beta-2 adrenergic receptors in a murine model reportedly increased the function of dermal fibroblasts and re-epithelization (37). In addition to their satisfactory survival in the present study, fibroblasts cultured on the mats also adhered to the nanoscaffold and showed favorable morphological characteristics. The elongated shape of fibroblasts on the PVA/PNL 4% mat was consistent with the morphological appearance of mature activated fibroblasts (38). 

In the present *in vivo* wound healing experiments, treatment with the PVA/PNL 4% mat was associated with better wound healing compared with other treatments in terms of macroscopic and especially microscopic parameters. Wounds treated with the PVA/PNL 4% mat had a higher rate of wound shrinkage and were the smallest in mean diameter compared with the other groups at different times. Wounds treated with PNL 4% gel also became smaller, with a wound shrinkage rate second only to PVA/PNL 4% mat-treated wounds. The superiority of PVA/PNL 4% mats in promoting wound healing was also evident when we assessed wound histopathological (microscopic) features. The fact that PNL-containing treatments were more effective in wound healing than non-PNL-containing treatments indicated the potential efficacy of PNL itself in promoting wound repair. However, as the PVA/PNL 4% mat proved to present a better healing profile than the PNL 4% gel, especially with respect to the microscopic wound healing and oxidative stress results, we may attribute the superiority of the PVA/PNL 4% mat to its nanofibrous structure which is lacking in the PNL 4% gel. The PVA/PNL 4% mat turned out to be highly porous (79.5 %). Unlike the gel formulation which lacks porosity, our porous wound dressing mat offers a high surface-to-volume ratio which potentially facilitates more cellular and molecular interactions with the nanofibers. Also, nanofibers have been reported to highly resemble natural extracellular matrix (20). The porous structure of nanofibers also allows more diffusion of air inward and exudates outward the wound bed providing a more dynamic wound healing medium. Nanofibers may also play a role as scaffolds to which the fibroblasts attach. This was clearly illustrated in the SEM image (see Figure 5b) in which stretched fibroblasts adhered onto the nanofibrous surface of the PVA/PNL 4% mat. In addition, tunability of drug release in nanofibers is another major advantage of them compared with traditional topical formulations such as gels. In this regard, we did not observe a burst release of PNL from the PVA/PNL 4% mat in the release test. Instead, the relatively sustained release of PNL from the fabricated mat potentially provided a longer presence of the drug in the wound medium leading to convincing wound healing. 

The promising potential of PNL in promoting wound healing has been well addressed in previous works. In clinical settings, PNL has been used successfully to treat infantile hemangiomas and the ulcerated form of this disease (3). Moreover, PNL and other beta-blockers were effective in wound healing by enhancing re-epithelization and keratinocyte migration in experimental studies (5, 8, 39). In light of the presence of beta-adrenergic receptors in the skin (8) and the detrimental effects of oxidative stress which might be induced by adrenergic activation, we studied markers of oxidative stress status (i.e., MDA and SOD) in an effort to provide a probable mechanistic explanation for our findings. Wounds treated with PNL-containing formulations, i.e., PNL 4% gel and especially the PVA/PNL 4% mat, resulted in less oxidative stress. As collagen production is highly vulnerable to oxidative stress in fibroblasts (40), at least part of the wound-healing effects of PNL may be associated with the lower oxidative stress in wound tissues. Figure 11 presents a schematic view of our proposed PNL mechanism in wound healing. Chronic overstimulation of beta-adrenergic receptors has been linked to increased ROS production probably through uncoupling of nitric oxide synthase (NOS) or other pathways (41). As previously mentioned, excess ROS detrimentally affects the functions of keratinocytes, fibroblasts, and other cells involved in wound healing. Hence, the illustration shows antagonism of these receptors on dermal cells by PNL might decrease oxidative stress and improve the wound healing process. However, characterizing the exact molecular mechanism of PNL in promoting wound healing requires further research with respect to the cross-talk between oxidative stress and other signaling cascades (42).

## Conclusion

Propanolol was successfully incorporated into nanofibers to yield a new pharmaceutical wound dressing mat with good physicochemical characteristics including thin fibers, high porosity, a good level of hydrophilicity, and sustained drug release. In addition, the PVA/PNL mat enhanced the viability of human cultured fibroblasts *in vitro*. The fabricated mat was also effective in accelerating wound healing in an animal model – an effect accompanied by significantly lower oxidative stress in the treated wounds, suggestive of a preliminary mechanism for the wound healing effects of PNL. We conclude that the potential biological effects of PNL on wound healing may be facilitated when this beta-blocker is used in a nanofiber-based wound dressing. The PVA/PNL mat described here may thus merit further investigation especially in clinical settings of wound repair. 

## Authors’ Contributions

SZ Study design and conception; FK, SZ Performing experiments; SZ Data analysis; FK Draft manuscript preparation; SZ Supervision and editing of the article; MA Funding acquisition and lab facilities; SZ, FK, and MA Final approval of the version to be published. 

## Conflicts of Interest

The authors declare that no conflict of interest exists.
